# A Photovoice exploration of youth vaping

**DOI:** 10.18332/popmed/215824

**Published:** 2025-12-31

**Authors:** Joy L. Hart, Kandi L. Walker, Alison C. McLeish, Savanna Kerstiens, Madeline M. Tomlinson, Lindsey A. Wood, Kolbie Vincent, Osayande Agbonlahor

**Affiliations:** 1 Department of Communication, College of Arts and Sciences, University of Louisville, Louisville, United States; 2 Christina Lee Brown Envirome Institute, School of Medicine, University of Louisville, Louisville, United States; 3 Department of Psychological and Brain Sciences, College of Arts and Sciences, University of Louisville, Louisville, United States; 4 School of Communication, Northwestern University, Evanston, United States; 5 College of Health, Psychology, and Social Care, University of Derby, Derby, United Kingdom; 6 Department of Epidemiology and Population Health, School of Public Health and Information Sciences, University of Louisville, Louisville, United States; 7 Department of Otolaryngology and Communicative Disorders, School of Medicine, University of Louisville, Louisville, United States; 8 Department of Preventive Medicine, University of Mississippi Medical Center, Jackson, United States

**Keywords:** electronic cigarettes, Photovoice, tobacco, vaping, youth

## Abstract

**INTRODUCTION:**

For over a decade, electronic cigarettes (e-cigarettes) have been the most used tobacco product among US youth. Although past research has stressed the importance of tailoring health communication messaging and interventions, much more work remains to be done in this area. To learn about youth views on vaping from their firsthand experience, we employed Photovoice to examine how youth experience vaping in their everyday lives.

**METHODS:**

Through Photovoice methodology and the SHOWED approach to discussion in 2022, we engaged US youth in conversation about vaping exposure and outcomes, focusing on how they experience vaping in their everyday lives. Employing the constant comparative method, discussion transcripts and notes were analyzed, revealing two overarching themes.

**RESULTS:**

Pervasiveness and Promotion, the first theme, illustrated the ever-present nature of vaping in the experiences of study participants. Health and Environmental Impacts, the second theme, described participants’ apprehensions regarding vaping’s negative effects.

**CONCLUSIONS:**

Overall, the examples, perspectives, and conclusions that the youth participants shared throughout these discussions illuminated frequent exposure to e-cigarette use and marketing as well as many concerns about the risks associated with use. Health communication messaging in these areas may be useful in refining existing and crafting new prevention and cessation campaigns.

## INTRODUCTION

Since their entry into the marketplace, electronic cigarettes (e-cigarettes) have become a popular tobacco consumption method among youth in the United States (US)^1,2^. In fact, by 2014, middle and high school student use of combustible cigarettes was eclipsed by their use of e-cigarettes, ranking the devices as the most common form of tobacco used by this age group^3^. The high level of use among young people led US Surgeon General Jerome Adams to declare a youth vaping epidemic in 2018^4^. Although youth use patterns have varied since that time, results from the 2024 US National Youth Tobacco Survey indicated that 5.9% of middle and high school students (i.e. approximately 1.63 million youth) reported past 30-day use of e-cigarettes and that e-cigarettes remained the most common tobacco product used by youth^2^. Given that numerous studies have documented the developmental and physiological harms of nicotine and tobacco product use among youth^3,5,6^, any level of tobacco consumption among youth is unsafe^3,7^. For example, nicotine consumption poses greater risks to young people as their brains are still developing^3^. Such risks include decreased impulse control, reduced cognition, diminished attention, and increased nicotine dependence.

Given the persistent e-cigarette marketing efforts directed toward youth^8^, the prevalence of youth use^2^, and the associated health risks, considerable attention has been devoted to youth vaping prevention efforts^9,10^, through avenues such as health campaigns (e.g. The Real Cost E-Cigarette Prevention Campaign^11^, designed to raise US teen awareness on the health dangers of vaping) and regulatory changes (e.g. Tobacco 21, legislation designed to curb youth tobacco use by raising the minimum legal age to purchase tobacco products in the US to 21 years^12^). Across this work, an often-overlooked perspective in e-cigarette use prevention and cessation efforts is that of youth themselves; in fact, calls to involve youth in such initiatives largely remain unheeded^13,14^. This omission results in missed opportunities to learn from youth who are at the vanguard of the youth vaping epidemic. The understanding gained through such engagement could result in health communication campaigns and prevention programs better adapted to youth and thus with higher potential for success.

Several possibilities exist for youth participation in work focused on improving health and preventing substance use, including efforts across multiple settings (e.g. schools and other organizations, families, social and legacy media, governmental structures and political processes, and communities^15^). Given the popularity of e-cigarettes among youth, some recent projects have worked to engage youth in initiatives to prevent or lessen youth vaping, such as peer-led support in the YES-CAN! Program^13^, building from their knowledge and experience of both the e-cigarette landscape in their communities and approaches that might resonate with their peers and others in their age group. Collaborative methods that rightly position youth as experts on their lived experience and encourage them to share their views and experiences with researchers are most likely to yield key insights. Youth describing firsthand how they experience e-cigarette availability, attitudes, and use among their peers and in their communities can contribute to fruitful discussions and ultimately advance scientific research, including development of youth-focused prevention and cessation programs. For example, insights gleaned from youth perspectives may contribute to program elements that are more relatable or have greater marketing reach.

Photovoice, a form of participatory photography, involves participants in taking photographs and then sharing their perspectives about the photographs^16,17^. These photos provide visual representations, making points easier to convey conversationally, especially for younger people, help to clarify, and serve to prompt information that might otherwise remain unaddressed^17^. Through sharing and discussing the photos, understanding grows. Considerable research supports using Photovoice and similar participatory photography methods with youth as pathways for them to share their lived experience^18^, and several studies have engaged youth in Photovoice projects, often in health and social change areas^19–21^. Through the sharing of lived experience via photographs and discussion of the photos, participants feel included in research processes and conclusions and researcher understanding is enhanced^20^. As researchers understand more about youth exposure to e-cigarettes in their daily environments and youth views of these products, researchers will be better positioned to develop key research questions as well as to contribute to prevention and cessation initiatives.

Across several health-related studies, Photovoice has been employed to engage youth, including in projects on tobacco^22–25^ and marijuana^26,27^ use among young people. Although these studies have differed in foci (e.g. physical and social environments, school smoking, tobacco and community health, gender and sexual identity, reasons for use and abstinence), agreement exists that Photovoice is a powerful and valuable method for engaging youth in reflection and dialogue, contributing to enhanced understanding of substance use and potential social change. However, despite the prevalence of e-cigarette use among youth and the declaration of a youth vaping epidemic, few studies using Photovoice to explore youth perspectives on vaping are available in the research literature. One notable exception is the META-Oak Project^28^, which examined youth perspectives on the marketing of e-cigarettes. This research gleaned insights into youth perceptions of e-cigarettes (e.g. seeing nonbiodegradable e-cigarette waste could generate curiosity), the appeal of marketing techniques (e.g. use of colors and flavors to attract youth attention), and communication surrounding these products (e.g. social media posting and trending). Despite the contributions of this project, vaping behavior was not examined, leaving many questions unaddressed and considerable work to be done. Another study that employed Photovoice to learn about student views of a university’s campus tobacco policy, included some findings related to e-cigarettes but was not limited to or focused on vaping products or behavior^29^, and this work involved college students, rather than middle and high school students.

To our knowledge, no published research examines using Photovoice with youth to explore vaping exposure and outcomes. To address this gap and learn about youth views of vaping from their firsthand experience, we employed Photovoice in the current study. More specifically, we asked youth participants to examine how they experience vaping in their everyday lives by taking photographs and participating in group discussions of participants’ photographs.

## METHODS

### Participants

Photovoice participants were members of the VapeRace Center’s Youth Advisory Council (YAC)^14^, a research center funded by the American Heart Association as part of its ENACT (i.e. End Nicotine Addiction in Children and Teens) initiative. Recruitment of participants took place through social media, middle and high school teacher networks, professional association contacts, and word of mouth across several geographical areas. During 2022, YAC members (n=22) participated in ‘VapePic’, which was designed to explore youth perspectives on vaping through photography. YAC members were aged 13–18 years (mean age=15.6 ± 1.62 years). Most reported female sex (63.6%), White race (81.8%), and being in 11th grade at school (45.5%). The university’s Institutional Review Board approved the study (protocol code #21.0372).

### Data collection

After parental consent, youth assent was obtained, and YAC members participated in two training sessions led by the study’s authors. These trainings included information on Photovoice objectives and methods, photography principles, and ethics (e.g. photographing only public behavior; not capturing identifying information, such as work badges, license plates, school or other uniforms, as well as faces and unique tattoos or piercings). Then, across several months, YAC members responded to question prompts by taking photos. Examples of question prompts included the following: ‘Where do you see vaping?’, ‘Who is vaping?’, and ‘What types of vaping messaging do you see?’. Participants were encouraged to take photos as part of their daily routines, rather than depart from their regular schedules or engage in new behavior to assess vaping. In short, the goal was to focus on their usual patterns or lived experience.

Given the wide accessibility of cell phones and that all members of YAC owned and regularly used one, youth took the photos (n=423) on their own devices in contexts where they commonly used them. Then, they sent these photos to study personnel for storage. During YAC meetings, the photos collected from the group served as starting points and prompts for discussion, with participants sharing information about one or two of their favorite photos and what the images conveyed or represented (n=52 shared). In these group discussions, YAC leaders facilitated a guided conversation based on the SHOWED approach, as described by Wallerstein and Bernstein^30^ for work in health contexts and modified by Wang^31^ for use in Photovoice applications. Using this approach, questions centered on what is depicted (i.e. ‘Seen’), occurring (i.e. ‘Happening’), the connection with others (i.e. ‘Our’), the cause (i.e. ‘Why’), the lesson (i.e. ‘Empowerment’), and the action needed (i.e. ‘Do’)^30,31^. After participants shared their photos and explored the context and meaning of these photos, the group discussed commonalities across the photos and recurrent points made in the conversation. The Photovoice sessions were recorded and transcribed, and notes were taken during the presentations and discussions.

### Analysis

Employing the constant comparative method^32–34^, two team members coded the data. This process began by reviewing meeting notes and transcripts for overall impressions. Then, these materials were read again, and annotations were made. The annotations noted key points, patterns, and potential categories, and then the coders compared and discussed these categories. Following the constant comparative method, themes were modified and refined as new information emerged^33^. From these discussions, recurrent themes were identified. Then, the coders re-read all materials (i.e. transcriptions and notes from Photovoice sessions, including the versions that each had annotated) and finalized agreement on the themes. As final steps, the themes were discussed among the coauthors as well as with several of the Photovoice participants^35^.

## RESULTS

During the Photovoice discussions, no participant reported having difficulty with identifying potential areas to take photos or what to photograph. Although they had previously shared information with the group about their exposure to vaping, these photos and their conversation when sharing the photos reinforced the commonplace nature of this exposure. Participants also described becoming even more aware of vaping behavior and imagery in their everyday lives than they had previously been prior to the Photovoice project. Even after completing the project, they continued to share examples that they had seen recently and related these examples to earlier Photovoice discussions.

Two overarching themes emerged from the analysis. The first theme, Pervasiveness and Promotion, underscored the ubiquitous nature of the youth vaping epidemic. For both e-cigarette use and e-cigarette marketing, the youth participants described how elements of each permeated much of their experience.

The second theme, Health and Environmental Impacts, conveyed participants’ concerns about the effects of vaping. Across the Photovoice discussions, participants frequently shared worries about the ways in which vaping might harm their peers’ health and how discarded vapes pose environmental risks.

Although participants frequently mentioned information in each of these thematic areas, the seemingly ever-present nature of e-cigarette messaging and use in their daily lives was discussed more often. Table 1 provides sample quotes for each theme.

## DISCUSSION

Through Photovoice, youth shared information about the ways in which they experience e-cigarettes in their daily lives. Past research has stressed the importance of understanding and including youth viewpoints, which will be helpful in developing improved health communication messaging and prevention and cessation interventions^36^. Despite its utility in engaging youth and conveying their experiences and insights, only one published Photovoice study has examined youth perspectives on e-cigarettes. This work, the META-Oak study^28^, focused solely on better understanding youth perspectives on the marketing of e-cigarette products to their age group. Our study expanded the focus to explore youth perspectives on use exposure and outcomes. Two themes emerged from the analysis of Photovoice discussion transcripts and notes. These themes are Pervasiveness and Promotion, which illustrated frequent exposure to both vaping and messages promoting vaping, and Health and Environmental Impacts, which revealed participant concerns about adverse effects to both humans and the environment.

### Pervasiveness and Promotion

The most prevalent theme, Pervasiveness and Promotion, highlighted the degree to which youth find e-cigarette use and messaging about e-cigarettes to be commonplace in their lives. Across the discussions, they emphasized that vaping and advertising about vaping was frequently encountered. Youth reported seeing vaping in cars, on school campuses (e.g. bathrooms, parking lots), outside of buildings, near food vendors, on sidewalks, at sport events, and at parties and other gatherings, among other places. Often, vapes were hidden up shirt sleeves, in pockets, or in other locations, and many vapes were made to resemble other items, such as computer flash drives, making them easier to conceal. Reflecting the views of many, one participant stated that, ‘It’s [vaping] everywhere. Everywhere you go. Everywhere you look’. Another shared that, ‘At my school, right across the street where every student goes during lunch break, tons of people smoke and vape in the alley that all students pass through every day’. Another commented on seeing users in cars blowing ‘large vape clouds’. Some discussion also centered on the location of vape shops, with participants, for example, showing photos and pointing out that these shops were often in easy to access locations (e.g. ‘next to a donut shop’, ‘by the pizza place’) where youth gather. One participant wondered why a fast-food restaurant taped a sign that read, ‘No smoking or vaping in the drive-thru’, on its pickup window. Another captured the views of the group by noting that e-cigarette use was ‘becoming too nonchalant and normalized’.

One participant noted the easy access to vaping information: ‘I went on Instagram and 30.8 million hashtags came up after a simple search with the word vape’. Beyond e-cigarettes online, these devices are also available at several places that youth frequently visit, such as convenience stores and gas stations. While sharing a photo of a gas station she regularly visits, one participant reported, ‘I occasionally stop at the Marathon for gas, and have noticed how there are multiple Marathons in my area which seem to be selling vapes to teenagers, disregarding the fact that they are underage, for it profits the store’. Participants shared additional photos of e-cigarette advertising and marketing and described this messaging with comments such as, ‘[Vape shop] is right next to my dance studio, and I pass it every Monday’. and ‘I saw at least five smoke shops with huge advertisements for e-cigarettes’. Another participant observed what she believed to be strategic placement of tobacco products in stores: ‘I thought it was interesting how all the cigarettes and vapes were advertised right next to the Mentos gum when you checkout’. Certainly, the ease of finding photo opportunities, the view that e-cigarette use is common, and perceptions of plentiful marketing and advertising of e-cigarettes, a point that aligns with findings in the META-Oak project^28^, indicate considerable youth exposure to these products, peers who use the products, and messaging encouraging such use.

### Health and Environmental Impacts

Given frequent exposure to e-cigarette use, perhaps it is not surprising that youth participants were concerned about the effects or outcomes of such use. The Health and Environmental Impacts theme foregrounded these concerns. Participants were apprehensive about the effects of e-cigarette use on their peers’ present and future health as well as on other groups (e.g. young adults that they frequently see featured in advertising or using the devices). Considerable discussion focused on not understanding why people vape as well as, conversely, acknowledging the power and danger of addiction. For example, one participant, while discussing her photo of a teen vaping while driving, stressed that, ‘Vaping is bad for your heart. I don’t understand why people do it, especially in a closed car’. Another described a picture of someone vaping while ‘wear(ing) a mask to protect her health from COVID-19’. When describing her photos of people vaping, one said that she finds herself thinking ‘I hope nothing bad happens to them’. Some discussion addressed schools taking action to discourage youth vaping. While describing a poster on a school bathroom door with the wording, ‘Crap belongs in the bowl, not in your lungs’, one participant shared the following: ‘When I was at school this week, I noticed they’re starting to take an initiative to stop teen vaping. I saw several posters advising students not to vape and how it poisons your body. Most of these posters are in the bathrooms, as that’s where vaping is most likely to occur’.

Beyond concerns about their peers’ health, the participants also expressed concern about environmental damage from e-cigarettes. Several participants shared photos that they had taken of vapes as trash on the ground and wondered about the risks to the environment. For example, one participant indicated that, ‘When it’s [a discarded e-cigarette] on the ground, I think about how bad it is for the environment and for the person’s health who used it’. Another observed that seeing discarded e-cigarette products on the ground was not unusual and that, in some places, ‘lots of these [e-cigarette products] were littered together’. The discussion included points about chemicals from e-cigarette waste being absorbed into soil or working its way into water systems as well as how long it might take plastic to degrade. In some cases, the participants connected their environmental concerns to human health concerns as well as effects on other animals.

Overall, the Photovoice experience illuminated considerable exposure to e-cigarette use and marketing among these youth. Given the pervasiveness of use and promotion of e-cigarette devices, participants regarded e-cigarette products as routine parts of daily life, suggesting that use and messaging have become normalized. Despite this regular exposure, these youth shared considerable concerns about health risks associated with e-cigarette use and environmental risks related to waste produced. Health communication messaging in these areas may enhance youth e-cigarette use prevention and cessation. Given their firsthand experience – and exposure, engaging youth in developing prevention and cessation messaging is important. Researchers can benefit from youth sharing their perspectives on ways to denormalize vaping among and effectively convey negative health and environmental impacts to their peers.

### Limitations

Our findings should be considered alongside several project limitations. First, the study participants were middle and high school students who worked together over the course of several months; thus, their exposure to youth vaping and viewpoints may not be generalizable to other youth. Second and relatedly, the participants were YAC members for a center dedicated to ending the youth vaping epidemic; thus, they may hold more informed and/or more negative views of vaping than non-YAC members. Third, recent declines in youth e-cigarette use have been reported^2^. Thus, although vape shops and store e-cigarette displays are still common in many locations, perhaps participant exposure to peer e-cigarette use would be somewhat lower if the Photovoice project were taking place today. Fourth, this research examined e-cigarette use and did not include use of other tobacco or cannabis products. Dual use of tobacco products (e.g. using e-cigarettes and combustible cigarettes) is frequent, and many US states have or are considering decriminalization and legalization of cannabis, both of which may influence youth views on e-cigarettes. However, these use patterns were not addressed in the current study. Fifth, our study included only US youth who self-selected into the project. It is possible that youth in other nations or without interest in projects such as this one have different experience and/or hold different views. Future work would benefit from examining other geographical contexts as well as more diverse samples of youth. Despite these limitations, this work makes several contributions to the literature, especially in including youth voices in research and given the scarcity of research on the use of Photovoice with youth regarding e-cigarettes. The findings of this project center youth participation in an important public health issue involving their age group – one with, as they point out, potential rippling effects for human and environmental health. In addition to informing future participatory research initiatives with youth, this work contributes to a growing body of literature on youth perceptions of vaping as well as offers insights in the realms of both policy and practice.

## CONCLUSIONS

Through Photovoice, these youth participants shared their experiences with and perspectives on youth e-cigarette use. As previous research has noted, engaging youth can deepen researcher understanding and has the potential to be useful in finetuning existing and developing new programs to curb youth vaping. Building from the adage, ‘A picture is worth a thousand words’, it becomes clear that Photovoice pictures and discussions are worth thousands upon thousands of words – vital ones in developing a clear image of the vaping epidemic through the eyes, camera lenses, and words of youth.

## Figures and Tables

**Table 1.** Themes and sample participant quotes, United States, 2022

Pervasiveness and Promotion	Health and Environmental Impacts
‘It’s [vaping] everywhere. Everywhere you go. Everywhere you look.’	‘When it’s [a discarded e-cigarette] on the ground, I think about how bad it is for the environment and for the person’s health who used it’
‘I went on Instagram and 30.8 million hashtags came up after a simple search with the word vape’	‘Vaping is bad for your heart. I don’t understand why people do it, especially in a closed car.’
At my school, right across the street where every student goes during lunch break, tons of people smoke and vape in the alley that all students pass through every day.’	‘When I was at school this week, I noticed they’re starting to take an initiative to stop teen vaping. I saw several posters advising students not to vape and how it poisons your body. Most of these posters are in the bathrooms, as that’s where vaping is most likely to occur’
‘[users in cars blowing] large vape clouds.’	‘You need to care about your health, like … this is a bad thing for you. This is not a joke’
‘I occasionally stop at the Marathon for gas, and have noticed how there are multiple Marathons in my area which seem to be selling vapes to teenagers, disregarding the fact that they are underage, for it profits the store.’	‘Vapes have become almost something of a casual accessory among teenagers, especially in social settings. Similar to having the newest phone or the newest sneakers, it’s something people notice and become fascinated with and get addicted to.’
‘[Vape shop] is right next to my dance studio, and I pass it every Monday.’	‘People toss them [vapes] on the ground. That creates a lot of trash, dangerous trash’
‘I saw at least five smoke shops with huge advertisements for e-cigarettes’	‘I see lots of vapes on the ground, and I wonder if their chemicals leak into the soil or water’
‘I thought it was interesting how all the cigarettes and vapes were advertised right next to the Mentos gum when you checkout’	‘I just don’t want to be around; [it’s] like secondhand smoke. I don’t … want to be breathing in the same areas … It feels like very chemical and just not good’
‘This sign shows how normalized vaping is, with the [vape] shop being located next to a tailor, a pizza shop, and a barber shop’	‘… vapes every time she gets out of school, like in her car. And so, I wonder how extreme the addiction is because it’s really sad’
‘… at lunch in the school cafeteria. He just came back from the bathroom where he got some “nic” from another student’	‘It’s their health and the health of people in the car with them, but it also damages the environment’
‘… on the [school] campus, it’s really bad. They’re like, people walk around [vaping], all the time on the way to class or whatnot.’	‘This is the parking lot. It’s bad that people just throw these [vapes] on the ground. But I also wonder if they are dangerous to the people who clean them up and when they are in landfills or wherever they go.’

## Data Availability

The data supporting this research are available from the authors on reasonable request.
